# Design, Synthesis, and Neuroprotective Effects of a Series of Pyrazolines against 6-Hydroxydopamine-Induced Oxidative Stress

**DOI:** 10.3390/molecules23092151

**Published:** 2018-08-27

**Authors:** Ahmet Özdemir, Belgin Sever, Mehlika Dilek Altıntop, Elif Kaya Tilki, Miriş Dikmen

**Affiliations:** 1 Department of Pharmaceutical Chemistry, Faculty of Pharmacy, Anadolu University, Eskişehir 26470, Turkey; belginsever@anadolu.edu.tr (B.S.); mdaltintop@anadolu.edu.tr (M.D.A.); 2 Department of Pharmacology, Faculty of Pharmacy, Anadolu University, Eskişehir 26470, Turkey; elif_kaya@anadolu.edu.tr (E.K.T.); mirisd@anadolu.edu.tr (M.D.)

**Keywords:** Parkinson’s disease, neurodegeneration, 2-pyrazoline, chalcone, 6-hydroxydopamine, pharmacokinetic parameters

## Abstract

Parkinson’s disease (PD) is a chronic, progressive, and age-related neurodegenerative disorder characterized by the loss of midbrain dopaminergic neurons caused by the accumulation of free radicals and oxidative stress. Based on the neuroprotective properties of 2-pyrazoline derivatives, in the current work, 1-(phenyl/4-substituted phenyl)-3-(2-furanyl/thienyl)-5-aryl-2-pyrazolines (**3a**–**i**, **4a**–**i**) were synthesized via the cyclization of the chalcones (**1**, **2**) with suitable phenylhydrazine hydrochloride derivatives. All these compounds were investigated for their neuroprotective effects using an in vitro 6-hydroxydopamine (6-OHDA)-induced neurotoxicity model of PD in the rat pheochromocytoma (PC-12) Adh cell line. In addition, some different pharmacokinetic parameters of all compounds were in silico predicted by the QikProp module of Schrödinger’s Maestro molecular modeling package. 4-Methylsulfonylphenyl substituted compounds **3h** (20%) and **4h** (23%) were determined as the most promising neuroprotective agents related to their inductive roles in cell viability when compared with the 6-OHDA-positive control group (43% and 42%, respectively). Moreover, in silico pharmacokinetic results indicated that all compounds were within the acceptable range intended for human use. According to both in vitro and in silico studies, compounds **3h** and **4h** draw attention as potential orally bioavailable therapeutic drug candidates against neurodegeneration in PD.

## 1. Introduction

Neurodegenerative diseases (NDDs) are rapidly rising in prevalence in many countries and are highly linked to the expected aging of the population, because these disorders mainly occur in the elderly. NDDs can be classified according to extrapyramidal and pyramidal movement problems or cognitive and behavioral problems. The most common forms of NDDs are Alzheimer’s disease (AD), Parkinson’s disease (PD), amyotrophic lateral sclerosis (ALS), Huntington’s disease (HD), and dementia with Lewy bodies (DLB) [1,2,3,4]. These diseases reveal common pathological properties such as the formation of insoluble protein-based aggregates in deteriorated neurons and glial cells, whereas there is a big difference in clinical features of patients suffering from them [5].

PD, firstly described as “paralysis agitans” by James Parkinson in 1817, is the second most common NDD affecting 2–3% of those >65 years of age [6,7]. The cause of PD is not clear, but the defects in mitochondrial functions and brain iron regulation, inflammation, and energy metabolism problems have been proven to be substantial in underlying mechanisms [8]. It is neuropathologically characterized by the loss of progressive dopaminergic neurons in the *substantia nigra pars compacta* and the accumulation of aggregated α-synuclein protein forming intracellular Lewy bodies in affected regions [8,9]. Moreover, the loss of dopaminergic neurons is associated with the decrease in the activity of catalase enzymes and the increase of monoamine oxidase-B (MAO-B) in glial cells. These enzymatic changes lead to emerging oxidative stress through the formation of higher levels of quinones, peroxides, and other reactive oxygen species (ROS), which then contribute to lipid and protein peroxidation and finally to neuronal death [8,9,10,11].

The cardinal clinical features of PD patients are motor impairment, including resting tremors, muscular rigidity, bradykinesia, and postural instability. Furthermore, these patients also mainly have speech and swallowing difficulties, a masklike facial expression, and micrographia [12,13]. Approved treatment procedures for PD to date, such as pharmacotherapy and neurosurgery, have mainly focused on the recovery of these clinical symptoms. However, recently a major approach for the treatment is to discover new beneficial, potential, and disease-modifying drugs targeting the underlying mechanisms related to the neurodegenerative process of PD [14,15,16].

**Figure 1:**
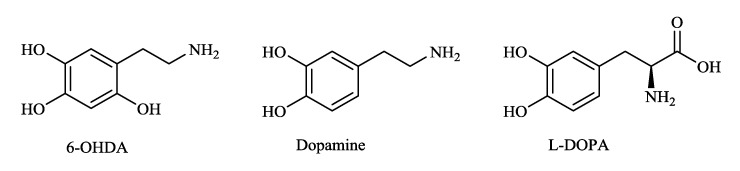
Chemical structures of 6-hydroxydopamine (6-OHDA), dopamine, and L-3,4-dihydroxyphenylalanine (L-DOPA).

The main aspect of oxidative stress in PD has been investigated with toxin-based models such as 1-methyl-4-phenyl-1,2,3,6-tetrahydropyridine (MPTP) and 6-hydroxydopamine (6-OHDA). 6-OHDA (Figure 1) is a neurotoxin, which is capable of generating ROS in neurons, and is detected in the urine of PD patients treated with long-term L-3,4-dihydroxyphenylalanine (L-DOPA) (Figure 1), which is the natural precursor of dopamine (Figure 1) and the typically prescribed drug for the symptomatic treatment of PD. Neurodegeneration with a 6-OHDA-based model can mimic idiopathic PD pathogenesis in vitro, as well as in vivo. The primary advantages of this model are measurable motor deficit (rotation) and proven usage in the pharmacological screening of compounds effective on dopamine and its receptors [17,18,19,20].

Rat pheochromocytoma (PC-12) cells have been widely used for in vitro studies exploring the mechanisms of NDDs. PC-12 Adh is a rat pheochromocytoma-derived cell-line, which responds to nerve growth factor (NGF) by switching from an immature chromaffin-cell-like phenotype to a sympathetic-neuron-like one. Therefore, this cell line has been widely used as an in vitro assay system for screening the neuroprotective effects of compounds [21,22,23,24].

Pyrazoline is a five-membered heterocyclic ring bearing two adjacent nitrogen atoms and one endocyclic double bond. It has three tautomeric forms called as 1-pyrazoline, 2-pyrazoline, and 3-pyrazoline, but among them, 2-pyrazoline is the most common one. 2-Pyrazoline-based compounds possess a wide range of pharmacological applications including antiepileptic, antidepressant, and anti-neurodegenerative activities. Moreover, these compounds can cross the blood–brain barrier (BBB) easily by means of their lipophilic characters [25,26,27,28].

Having the above aspects in mind, a series of 2-pyrazoline-based compounds **3a**–**i** and **4a**–**i** were designed and synthesized via the reaction of chalcones (**1**, **2**). All the compounds were in vitro evaluted for their neuroprotective potentials against 6-OHDA-induced neurotoxicity in PC-12 Adh cells. In addition, in silico absorption, distribution, metabolism, and excretion (ADME) studies were performed to predict their physicochemical properties particularly their abilities to cross the BBB.

## 2. Results and Discussion

**Scheme 1:**
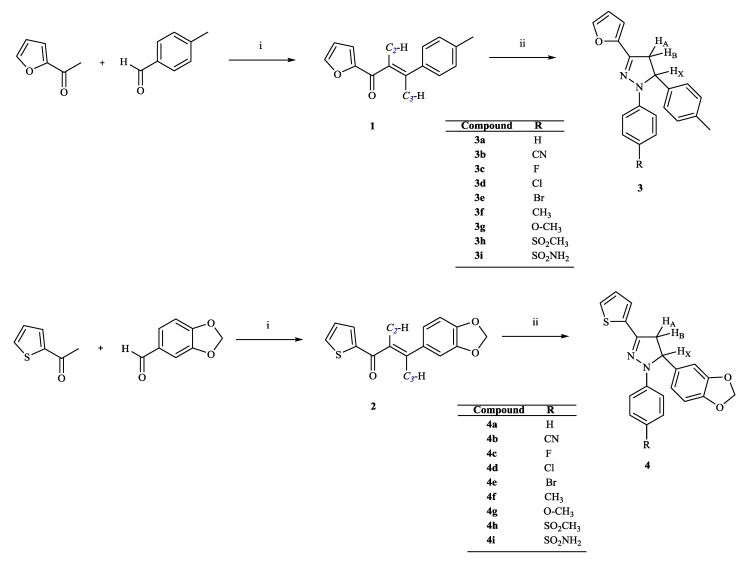
The synthetic route for the preparation of the compounds (**1**, **2**, **3a**–**i**, **4a**–**i**). Reagents and conditions: (i) 40% (*w*/*v*) sodium hydroxide, ethanol, rt, 24 h; (ii) appropriate phenylhydrazine hydrochloride derivative, CH_3_COOH, reflux, 8 h.

The synthesis of new pyrazoline derivatives (**3a**–**i**, **4a**–**i**) followed the general pathway depicted in Scheme 1. Initially, 1,3-diaryl-substituted chalcones (**1**, **2**) were synthesized via the base-catalyzed Claisen–Schmidt condensation of 2-acetylfuran/2-acetylthiophene with the appropriate aromatic aldehydes [29,30,31,32]. Then, the final compounds, 1-(phenyl/4-substituted phenyl)-3-(2-furanyl/thienyl)-5-aryl-2-pyrazolines (**3a**–**i**, **4a**–**i**), were obtained via the cyclization of the chalcones (**1**, **2**) with suitable phenylhydrazine hydrochloride derivatives in the presence of hot acetic acid [33].

The structures of all the compounds were elucidated by FTIR, ^1^H NMR, ^13^C NMR, mass spectral data, and elemental analyses. In the ^1^H NMR spectra of the compounds, **3a**–**i** and **4a**–**i**, the CH_2_ protons of the 2-pyrazoline ring resonated as a pair of doublets of doublets at 2.95–3.20 ppm (H_A_) (*J_AM_* = 17.13–17.64 Hz, *J_AX_* = 4.65–7.41 Hz) and 3.69–3.96 ppm (H_M_) (*J_MA_* = 17.18–17.64 Hz, *J_MX_* = 11.85–12.09 Hz). The CH proton appeared as a doublet of doublets at 5.29–5.59 ppm (H_X_) (*J_MX_* = 11.76–12.09 Hz, *J_AX_* = 4.68–7.38 Hz) owing to the vicinal coupling with two magnetically non-equivalent protons of the methylene group at the 4th position of the pyrazoline ring. All the other aromatic and aliphatic protons were observed in the expected regions. Mass spectral data and elemental analysis were also in agreement with the proposed structures of the compounds [33,34].

**Figure 2:**
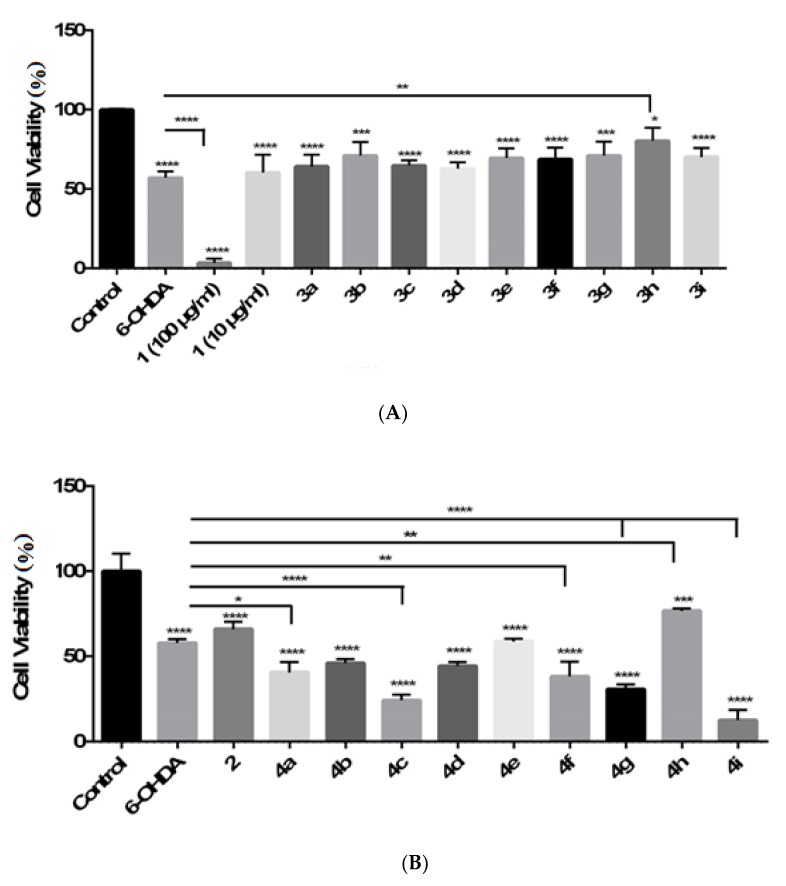
Neuroprotective effects of compounds **1** and **3a**–**i** (**A**); **2** and **4a**–**i** (**B**) against 6-OHDA induced neurotoxicity. PC-12 Adh cells were treated with 100 µg/mL concentration of the compounds for 6 h before exposure to 150 µM 6-OHDA for 24 h. The cell viability was detected at the 24th hour. The results of the cell viability were presented as a % of the control (the O.D. value). Data are shown as mean ± SD of three experiments. ((*n* = 8), *p* > 0.05 n.s., * *p* < 0.05, ** *p* < 0.01, *** *p* < 0.001, and **** *p* < 0.0001).

According to the IC_50_ values given in Table 1, apart from compound **1**, treatment with the compounds at 100 μg/mL did not reduce cell viability significantly. The compounds demonstrated their protective effects against cell death in differentiated PC-12 Adh cells treated with 6-OHDA (150 μM) for 24 h. The reduction in cell viability was determined as 43% with 6-OHDA (150 μM) and 36, 29, 35, 37, 30, 31, 29, 20, 30% with compounds **3a**–**i** (100 µg/mL), respectively (Figure 2; Table 2). However, compound **1** was found to be highly cytotoxic, and it reduced cell viability with the values of 97% (100 µg/mL) and 40% (10 µg/mL). In addition, the reduction in cell viability was evaluated as 34.1% with compound **2** and 59, 54, 76, 56, 41, 62, 69, 23, 87% with compounds **4a**–**i** (100 µg/mL), respectively, when compared with 6-OHDA (150 μM) (42.13%) (Figure 2; Table 2). According to these results, all the compounds reduced cell viability compared with the control group after 24 h 6-OHDA exposure (* *p* < 0.05, *** *p* < 0.001, **** *p* < 0.0001 for compounds **1** and **3a**–**i**; **** *p* < 0.0001 for compounds **2** and **4a**–**i**). Compounds **3a**–**i**, **2**, and **4h** might have neuroprotective potential against 6-OHDA induced neurotoxicity in PC-12 Adh cells but only the induction in cell viability percentage with compounds **3h** and **4h** was found significant compared with the 6-OHDA-positive control group related to the statistical analysis (** *p* < 0.01). This outcome pointed out that the presence of 4-methylsulfonylphenyl moiety enhanced the neuroprotective potency of the 2-pyrazoline ring as observed in both compounds **3h** and **4h**.

ADME properties of compounds **1**, **2**, **3a**–**i**, and **4a**–**i** were in silico assessed to enlighten the biological, pharmaceutical, and drug similarities of these compounds. The results given in Table 3 were found to be within the acceptable range intended for human use, making these derivatives promising drug candidates [35]. All these compounds were detected to exhibit excellent absorption % (92–100%) in human oral absorption on a 0–100% scale, and based on the parameters of the brain/blood partition coefficient (QPlogBB) (−1.08 to 0.78) and central nervous system (CNS) activity (−1 to 0), they were also found to pass through the BBB, which restrains drug entry from blood into brain by multiple mechanisms [36,37]. Solvent accessible surface area (SASA) is used to define the accessibility of the residue to the solvent; either it is between lipid or water accessibility, and it is essential to BBB permeability [38]. The SASA values of all the compounds were found within the range (467–680). The results also indicated that all the compounds obey Lipinski’s rule of five and Jorgensen’s rule of three; due to the fact that a potential orally active drug candidate should reveal no more than one violation of these rules [39,40].

## 3. Materials and Methods

### 3.1. Chemistry

All reagents were purchased from commercial suppliers and were used without further purification. Melting points (M.p.) were determined on an Electrothermal 9100 melting point apparatus (Weiss-Gallenkamp, Loughborough, UK) and are uncorrected. IR spectra were recorded on an IRPrestige-21 Fourier Transform Infrared spectrophotometer (Shimadzu, Tokyo, Japan). ^1^H NMR and ^13^C NMR spectra were recorded by a Bruker digital FT-NMR spectrometer (Bruker Bioscience, Billerica, MA, USA) in DMSO-*d*_6_. Mass spectra were recorded on an Agilent LC-MSD-Trap-SL Mass spectrometer (Agilent Technologies, Palo Alto, CA, USA). Elemental analyses were performed on a Perkin Elmer EAL 240 elemental analyzer (Perkin-Elmer, Norwalk, CT, USA) and the results were within ±0.4% of the theoretical values. Thin Layer Chromatography (TLC) was performed on TLC Silica gel 60 F254 aluminium sheets (Merck, Darmstadt, Germany) to check the purity of the compounds.

#### 3.1.1. General Procedure for the Synthesis of the Compounds

##### 1-(2-Furanyl/thienyl)-3-aryl-2-propen-1-one (**1**, **2**)

2-Acetylfuran/2-acetylthiophene (0.02 mol), proper aromatic aldehyde (0.02 mol) and 40% (*w*/*v*) sodium hydroxide (5 mL) in ethanol (30 mL) were stirred at room temperature for 24 h. Then, the reaction mixture was poured into ice and the precipitated solid was filtered, washed with water, and dried. The product was crystallized from ethanol [29,30,31,32,33,34].

*1-(2-Furanyl)-3-aryl-2-propen-1-one* (**1**). Yield: 55%. M.p.: 111–113 °C. Lit. M.p.: 96–98 °C [41]. IR ν_max_ (cm^−^^1^): 3126.61 (aromatic C-H stretching), 2927.94 (aliphatic C-H stretching), 1651.07 (C=O stretching), 1597.06, 1558.48, 1512.19, 1462.04 (C=C stretching), 1411.89, 1392.61, 1332.81, 1300.02, 1288.45, 1247.94, 1207.44, 1182.36, 1161.15, 1083.99, 1053.13, 1010.70 (C-O stretching and aromatic C-H in plane bending), 981.77, 927.76, 883.40, 864.11, 842.89, 813.96, 802.39, 765.74, 727.16 (aromatic C-H out of plane bending). ^1^H-NMR (300 MHz, DMSO-*d*_6_) δ (ppm): 2.35 (3H, s, CH_3_), 6.79 (1H, dd, *J* = 3.57 Hz, 1.71 Hz aromatic proton), 7.28 (2H, d, *J* = 7.92 Hz, aromatic protons), 7.65 (1H, d, *J* = 15.72 Hz, C_3_-H), 7.70 (1H, d, *J* = 8.76 Hz, aromatic proton), 7.74–7.76 (2H, m, C_2_-H and aromatic proton), 7.81 (1H, dd, *J* = 3.60 Hz, 0.63 Hz, aromatic proton), 8.07 (1H, dd, *J* = 1.65 Hz, 0.66 Hz, aromatic proton). ^13^C-NMR (75 MHz, DMSO-*d*_6_) δ (ppm): 21.54 (CH_3_), 113.16 (CH), 119.79 (CH), 121.37 (C_2_-H), 129.52 (2CH), 130.03 (2CH), 132.19 (C), 141.20 (C), 143.30 (C_3_-H), 148.74 (CH), 153.46 (C), 177.15 (C, C=O). Anal. Calcd. for C_14_H_12_O_3_: C, 79.22; H, 5.70; Found: C, 79.12; H, 5.64. MS (ESI) (*m/z*): [M + H]^+^ 212.08.

*1-(2-Thienyl)-3-aryl-2-propen-1-one* (**2**). Yield: 60%. M.p.: 126–128 °C. Lit. M.p.: 117–119 °C [35,42]. IR ν_max_ (cm^−^^1^): 3105.39 (aromatic C-H stretching), 2989.66, 2906.73 (aliphatic C-H stretching), 1643.55 (C=O stretching), 1604.77, 1583.56, 1498.69, 1487.12, 1444.68 (C=C stretching), 1411.89, 1369.46, 1346.31, 1305.81, 1246.02, 1228.66, 1215.15, 1192.01, 1105.21, 1058.92, 1028.06 (C-O stretching and aromatic C-H in plane bending), 972.12, 925.83, 916.19, 860.25, 831.32, 800.46, 727.16, 700.16 (aromatic C-H out of plane bending and C-S stretching). ^1^H-NMR (300 MHz, DMSO-*d*_6_) δ (ppm): 6.12 (2H, s, O-CH_2_-O), 7.00 (1H, d, *J* = 8.01 Hz, aromatic proton), 7.30–7.36 (2H, m, aromatic protons), 7.64-7.69 (2H, m, C_2_-H and aromatic protons), 7.76 (1H, d, *J* = 15.51 Hz, C_3_-H), 8.04 (1H, dd, *J* = 4.92 Hz, 0.99 Hz, aromatic proton), 8.32 (1H, dd, *J* = 3.78 Hz, 0.99 Hz, aromatic proton). ^13^C-NMR (75 MHz, DMSO-*d*_6_) δ (ppm): 102.15 (CH_2_), 107.42 (CH), 109.01 (CH), 120.35 (CH), 126.46 (C_2_-H), 129.29 (CH), 129.50 (C), 133.83 (CH), 135.70 (CH), 143.61 (C_3_-H), 146.26 (C), 148.58 (C), 150.10 (C), 182.00 (C, C=O). Anal. Calcd. for C_14_H_10_O_3_S: C, 65.10; H, 3.90; Found: C, 64.98; H, 3.84. MS (ESI) (*m/z*): [M + H]^+^ 258.04.

##### 1-(Phenyl/4-Substituted phenyl)-3-(2-furanyl/thienyl)-5-aryl-2-pyrazolines (**3a**–**i**, **4a**–**i**)

A mixture of appropriate chalcone (**1**, **2**) (10.0 mmol) and phenylhydrazine hydrochloride derivative (20.0 mmol) was refluxed for 8 h in absolute ethanol (30 mL) in the presence of acetic acid (10 mL) to get 2-pyrazolines. Then the reaction mixture was poured into crushed ice. The precipitate was separated by filtration, washed with water and crystallized from methanol [33].

*1-Phenyl-3-(2-furanyl)-5-(4-methylphenyl)-2-pyrazoline* (**3a**) [43]. Yield: 67%. M.p.: 162–163 °C. IR ν_max_ (cm^−^^1^): 3113.11, 3034.03 (aromatic C-H stretching), 2918.30, 2848.86 (aliphatic C-H stretching), 1595.13, 1498.69, 1487.12 (C=N and C=C stretching), 1413.82, 1365.60, 1334.74, 1319.31, 1247.94, 1176.58, 1128.36, 1072.42, 1051.20, 1033.85, 1022.27 (C-N, C-O stretching and aromatic C-H in plane bending), 997.20, 960.55, 923.90, 885.33, 869.90, 842.89, 813.96, 744.52, 731.02, 690.52, 673.16 (aromatic C-H out of plane bending). ^1^H-NMR (300 MHz, DMSO-*d*_6_) δ (ppm): 2.25 (3H, s, CH_3_), 2.98 (1H, dd, *J_AM_* = 17.28 Hz, *J_AX_* = 6.15 Hz, C_4_-H_A_ pyrazoline), 3.82 (1H, dd, *J_MA_* = 17.28 Hz, *J_MX_* = 12.12 Hz, C_4_-H_M_ pyrazoline), 5.40 (1H, dd, *J_MX_* = 12.09 Hz, *J_AX_* = 6.12 Hz, C_5_-H_X_ pyrazoline), 6.61 (1H, dd, *J* = 3.42 Hz, 1.80 Hz, aromatic proton), 6.68–6.77 (2H, m, aromatic protons), 6.95 (2H, d, *J* = 8.72 Hz, aromatic protons), 7.10–7.16 (6H, m, aromatic protons), 7.80 (1H, dd, *J* = 1.71 Hz, 0.63 Hz). ^13^C-NMR (75 MHz, DMSO-*d*_6_) δ (ppm): 22.11 (CH_3_), 43.39 (CH_2_), 62.85 (CH), 111.12 (CH), 112.42 (CH), 113.45 (2CH), 119.05 (CH), 126.22 (2CH), 129.30 (2CH), 129.66 (C), 130.01 (2CH), 137.08 (CH), 139.66 (C, d, *J* = 3.75 Hz), 144.62 (2C, d, *J* = 6.00 Hz), 147.97 (C). Anal. Calcd. for C_20_H_18_N_2_O: C, 79.44; H, 6.00; N, 9.26; Found: C, 79.39; H, 6.04; N, 9.25. MS (ESI) (*m/z*): [M]^+^ 302.00, [M + H]^+^ 303.00.

*1-(4-Cyanophenyl)-3-(2-furanyl)-5-(4-methylphenyl)-2-pyrazoline* (**3b**). Yield: 55%. M.p.: 77–78 °C. IR ν_max_ (cm^−^^1^): 3118.90 (aromatic C-H stretching), 2918.30 (aliphatic C-H stretching), 2212.35 (C≡N stretching), 1598.99, 1510.26 (C=N and C=C stretching), 1415.75, 1373.32, 1323.17, 1174.65, 1122.57, 1004.91 (C-N, C-O stretching and aromatic C-H in plane bending), 812.03, 740.67 (aromatic C-H out of plane bending). ^1^H-NMR (300 MHz, DMSO-*d*_6_) δ (ppm): 2.25 (3H, s, CH_3_), 3.06 (1H, dd, *J_AM_* = 17.58 Hz, *J_AX_* = 4.65 Hz, C_4_-H_A_ pyrazoline), 3.90 (1H, dd, *J_MA_* = 17.49 Hz, *J_MX_* = 11.91 Hz, C_4_-H_M_ pyrazoline), 5.58 (1H, dd, *J_MX_* = 11.76 Hz, *J_AX_* = 4.68 Hz, C_5_-H_X_ pyrazoline), 6.64–6.65 (1H, m, aromatic proton), 6.90 (1H, d, *J* = 3.30 Hz, aromatic proton), 7.01 (2H, d, *J* = 8.73 Hz, aromatic protons), 7.07–7.16 (4H, m, aromatic protons), 7.54 (2H, d, *J* = 8.73 Hz, aromatic protons), 7.86 (1H, s, aromatic proton). ^13^C-NMR (75 MHz, DMSO-*d*_6_) δ (ppm): 21.10 (CH_3_), 43.44 (CH_2_), 61.91 (CH), 99.40 (C), 112.66 (CH), 112.98 (CH), 113.25 (2CH), 120.39 (C), 126.04 (2CH), 130.19 (2CH), 133.76 (2CH), 137.50 (C), 138.56 (C), 142.89 (2C), 145.53 (CH), 146.89 (C). Anal. Calcd. for C_21_H_17_N_3_O: C, 77.04; H, 5.23; N, 12.84; Found: C, 77.06; H, 5.24; N, 12.82. MS (ESI) (*m/z*): [M + H]^+^ 327.90.

*1-(4-Fluorophenyl)-3-(2-furanyl)-5-(4-methylphenyl)-2-pyrazoline* (**3c**). Yield: 53%. M.p.: 87–89 °C. IR ν_max_ (cm^−^^1^): 2987.74, 2900.94 (aliphatic C-H stretching), 1597.06, 1508.33 (C=N and C=C stretching), 1413.82, 1373.32, 1321.24, 1172.72, 1002.98 (C-N, C-O stretching and aromatic C-H in plane bending), 812.03, 742.59 (aromatic C-H out of plane bending). ^1^H-NMR (300 MHz, DMSO-*d*_6_) δ (ppm): 2.25 (3H, s, CH_3_), 2.98 (1H, dd, *J_AM_* = 17.25 Hz, *J_AX_* = 6.57 Hz, C_4_-H_A_ pyrazoline), 3.82 (1H, dd, *J_MA_* = 17.25 Hz, *J_MX_* = 12.03 Hz, C_4_-H_M_ pyrazoline), 5.37 (1H, dd, *J_MX_* = 11.97 Hz, *J_AX_* = 6.54 Hz, C_5_-H_X_ pyrazoline), 6.61 (1H, dd, *J* = 3.36 Hz, 1.77 Hz, aromatic proton), 6.76 (1H, d, *J* = 3.33 Hz, aromatic proton), 6.93–6.99 (4H, m, aromatic protons), 7.15 (4H, s, aromatic protons), 7.80 (1H, d, *J* = 1.44 Hz, aromatic proton). ^13^C-NMR (75 MHz, DMSO-*d*_6_) δ (ppm): 21.09 (CH_3_), 43.57 (CH_2_), 63.42 (CH), 111.20 (CH), 112.43 (CH), 114.65 (CH, d, *J* = 7.5 Hz), 115.84 (CH, d, *J* = 22.5 Hz), 126.32 (2CH), 130.04 (2CH), 137.18 (CH), 139.36 (CH), 139.93 (C), 141.56 (C), 144.70 (CH), 147.56 (C), 147.88 (C), 154.79 (C), 157.90 (C). Anal. Calcd. for C_20_H_17_FN_2_O: C, 74.98; H, 5.35; N, 8.74; Found: C, 74.94; H, 5.33; N, 8.76. MS (ESI) (*m/z*): [M + H]^−^ 318.90, [M]^+^ 320.90, [M + H]^+^ 321.90.

*1-(4-Chlorophenyl)-3-(2-furanyl)-5-(4-methylphenyl)-2-pyrazoline* (**3d**). Yield: 83%. M.p.: 115–117 °C. IR ν_max_ (cm^−^^1^): 2987.74, 2900.94 (aliphatic C-H stretching), 1598.99, 1492.90 (C=N and C=C stretching), 1375.25, 1076.28, 1051.20 (C-N, C-O stretching and aromatic C-H in plane bending), 817.82, 734.88 (aromatic C-H out of plane bending). ^1^H-NMR (300 MHz, DMSO-*d*_6_) δ (ppm): 2.25 (3H, s, CH_3_), 3.00 (1H, dd, *J_AM_* = 17.37 Hz, *J_AX_* = 5.85 Hz, C_4_-H_A_ pyrazoline), 3.84 (1H, dd, *J_MA_* = 17.37 Hz, *J_MX_* = 12.06 Hz, C_4_-H_M_ pyrazoline), 5.43 (1H, dd, *J_MX_* = 12.06 Hz, *J_AX_* = 5.82 Hz, C_5_-H_X_ pyrazoline), 6.61 (1H, dd, *J* = 3.45 Hz, 1.80 Hz, aromatic proton), 6.79 (1H, d, *J* = 3.93 Hz, aromatic proton), 6.93 (2H, d, *J* = 7.94 Hz, aromatic protons), 7.13 (4H, s, aromatic protons), 7.17 (2H, d, *J* = 9.00 Hz, aromatic protons), 7.81 (1H, dd, *J* = 1.68 Hz, 0.63 Hz, aromatic proton). ^13^C-NMR (75 MHz, DMSO-*d*_6_) δ (ppm): 21.08 (CH_3_), 43.49 (CH_2_), 62.75 (CH), 111.60 (CH), 112.48 (CH), 114.85 (2CH), 122.60 (C), 126.20 (2CH), 129.11 (2CH), 130.07 (2CH), 137.23 (C), 139.11 (C), 140.51 (C), 143.36 (C), 144.89 (CH), 147.73 (C). Anal. Calcd. for C_20_H_17_ClN_2_O: C, 71.32; H, 5.09; N, 8.32; Found: C, 71.28; H, 5.03; N, 8.35. MS (ESI) (*m/z*): [M + H]^−−^ 334.90, [M]^+^ 336.90, [M + H]^+^ 337.90.

*1-(4-Bromophenyl)-3-(2-furanyl)-5-(4-methylphenyl)-2-pyrazoline* (**3e**). Yield: 79%. M.p.: 133–135 °C. IR ν_max_ (cm^−^^1^): 2999.31, 2920.23 (aliphatic C-H stretching), 1593.20, 1512.19, 1492.90, 1483.26 (C=N and C=C stretching), 1365.60, 1319.31, 1122.57, 1087.85, 1074.35, 1051.20, 1004.91 (C-N, C-O stretching and aromatic C-H in plane bending), 883.40, 862.18, 815.89, 792.74, 732.95 (aromatic C-H out of plane bending). ^1^H-NMR (300 MHz, DMSO-*d*_6_) δ (ppm): 2.25 (3H, s, CH_3_), 3.00 (1H, dd, *J_AM_* = 17.37 Hz, *J_AX_* = 5.76 Hz, C_4_-H_A_ pyrazoline), 3.83 (1H, dd, *J_MA_* = 17.34 Hz, *J_MX_* = 12.06 Hz, C_4_-H_M_ pyrazoline), 5.43 (1H, dd, *J_MX_* = 12.03 Hz, *J_AX_* = 5.70 Hz, C_5_-H_X_ pyrazoline), 6.61 (1H, dd, *J* = 3.42 Hz, 1.80 Hz, aromatic proton), 6.79 (1H, d, *J* = 3.39 Hz, aromatic proton), 6.88 (2H, d, *J* = 9.00 Hz, aromatic protons), 7.12 (4H, s, aromatic protons), 7.29 (2H, d, *J* = 8.97 Hz, aromatic protons), 7.81 (1H, t, *J* = 1.65 Hz, 1.08 Hz, 0.57 Hz, aromatic proton). ^13^C-NMR (75 MHz, DMSO-*d*_6_) δ (ppm): 21.09 (CH_3_), 43.48 (CH_2_), 62.64 (CH), 110.21 (C), 111.65 (CH), 112.49 (CH), 115.35 (2CH), 126.19 (2CH), 130.07 (2CH), 131.94 (2CH), 137.24 (C), 139.05 (C), 140.57 (C), 143.67 (C), 144.91 (CH), 147.72 (C). Anal. Calcd. for C_20_H_17_BrN_2_O: C, 63.00; H, 4.49; N, 7.35; Found: C, 63.04; H, 4.53; N, 7.32. MS (ESI) (*m/z*): [M + H]^−−^ 378.80, [M]^+^ 380.80, [M + H]^+^ 381.90, [M + H]^+++^ 383.00.

*1-(4-Methylphenyl)-3-(2-furanyl)-5-(4-methylphenyl)-2-pyrazoline* (**3f**). Yield: 53%; M.p. 67–68 °C. IR ν_max_ (cm^−^^1^): 2972.31, 2900.94 (aliphatic C-H stretching), 1614.42, 1512.19 (C=N and C=C stretching), 1408.04, 1365.60, 1249.87, 1074.35, 1049.28 (C-N, C-O stretching and aromatic C-H in plane bending), 883.40, 804.32, 738.74 (aromatic C-H out of plane bending). ^1^H-NMR (300 MHz, DMSO-*d*_6_) δ (ppm): 2.15 (3H, s, CH_3_), 2.24 (3H, s, CH_3_), 2.95 (1H, dd, *J_AM_* = 17.19 Hz, *J_AX_* = 6.36 Hz, C_4_-H_A_ pyrazoline), 3.79 (1H, dd, *J_MA_* = 17.22 Hz, *J_MX_* = 12.09 Hz, C_4_-H_M_ pyrazoline), 5.36 (1H, dd, *J_MX_* = 12.09 Hz, *J_AX_* = 6.33 Hz, C_5_-H_X_ pyrazoline), 6.60 (1H, dd, *J* = 3.42 Hz, 1.80 Hz, aromatic proton), 6.72–6.74 (1H, m, aromatic proton), 6.83–6.86 (2H, m, aromatic protons), 6.94 (2H, d, *J* = 8.34 Hz, aromatic protons), 7.09–7.13 (4H, m, aromatic protons), 7.78 (1H, dd, *J* = 1.71 Hz, 0.63 Hz, aromatic proton). ^13^C-NMR (75 MHz, DMSO-*d*_6_) δ (ppm): 20.54 (CH_3_), 21.08 (CH_3_), 43.34 (CH_2_), 63.13 (CH), 110.79 (CH), 112.39 (CH), 113.63 (2CH), 126.28 (2CH), 127.76 (CH), 128.80 (C), 129.73 (2CH, d, *J* = 16.50 Hz), 137.00 (C), 139.16 (C), 139.67 (CH), 142.51 (2C), 144.51 (CH), 148.08 (C). Anal. Calcd. for C_21_H_20_N_2_O: C, 79.72; H, 6.37; N, 8.85; Found: C, 79.66; H, 6.28; N, 8.89. MS (ESI) (*m/z*): [M]^+^ 316.90, [M + H]^+^ 318.00.

*1-(4-Methoxyphenyl)-3-(2-furanyl)-5-(4-methylphenyl)-2-pyrazoline* (**3g**). Yield: 27%; M.p. 70–72 °C. IR ν_max_ (cm^−^^1^): 3118.90 (aromatic C-H stretching), 2918.30, 2831.50 (aliphatic C-H stretching), 1606.70, 1568.13, 1506.41, 1463.97 (C=N and C=C stretching), 1359.82, 1292.31, 1238.30, 1178.51, 1120.64, 1097.50, 1035.77 (C-N, C-O stretching and aromatic C-H in plane bending), 883.40, 873.75, 815.89, 746.45 (aromatic C-H out of plane bending). ^1^H-NMR (300 MHz, DMSO-*d*_6_) δ (ppm): 2.25 (3H, s, CH_3_), 2.95 (1H, dd, *J_AM_* = 17.13 Hz, *J_AX_* = 7.14 Hz, C_4_-H_A_ pyrazoline), 3.63 (3H, s, OCH_3_), 3.69 (1H, dd, *J_MA_* = 17.18 Hz, *J_MX_* = 12.00 Hz, C_4_-H_M_ pyrazoline), 5.30 (1H, dd, *J_MX_* = 11.97 Hz, *J_AX_* = 7.17 Hz, C_5_-H_X_ pyrazoline), 6.59 (1H, dd, *J* = 3.42 Hz, 1.80 Hz, aromatic proton), 6.71–6.77 (3H, m, aromatic protons), 6.86–6.90 (2H, m, aromatic protons), 7.12–7.18 (4H, m, aromatic protons), 7.78 (1H, dd, *J* = 1.71 Hz, 0.66 Hz, aromatic proton). ^13^C-NMR (75 MHz, DMSO-*d*_6_) δ (ppm): 21.08 (CH_3_), 43.47 (CH_2_), 55.63 (CH_3_), 63.94 (CH), 106.80 (CH), 110.58 (CH), 112.36 (CH), 114.88 (2CH, d, *J* = 12.75 Hz), 126.42 (2CH), 127.34 (C), 130.01 (2CH, d, *J*= 8.25 Hz), 137.03 (C), 138.97 (C), 139.16 (C), 139.69 (CH), 144.41 (CH), 148.14 (C), 153.15 (C). Anal. Calcd. for C_21_H_20_N_2_O_2_: C, 75.88; H, 6.06; N, 8.43; Found: C, 75.90; H, 6.04; N, 8.50. MS (ESI) (*m/z*): [M]^+^ 332.00, [M + H]^+^ 333.00.

*1-(4-Methylsulfonylphenyl)-3-(2-furanyl)-5-(4-methylphenyl)-2-pyrazoline* (**3h**). Yield: 68%. M.p.: 162–163 °C. IR ν_max_ (cm^−^^1^): 2985.81, 2900.94 (aliphatic C-H stretching), 1589.34, 1506.41 (C=N and C=C stretching), 1406.11, 1379.10, 1292.31, 1242.16, 1132.21, 1066.64 (C-N, C-O stretching and aromatic C-H in plane bending), 960.55, 883.40, 815.89, 769.60 (aromatic C-H out of plane bending and C-S stretching). ^1^H-NMR (300 MHz, DMSO-*d*_6_) δ (ppm): 2.24 (3H, s, CH_3_), 3.00 (1H, dd, *J_AM_* = 17.37 Hz, *J_AX_* = 5.76 Hz, C_4_-H_A_ pyrazoline), 3.06 (3H, s, SO_2_CH_3_), 3.91 (1H, dd, *J_MA_* = 17.55 Hz, *J_MX_* = 11.97 Hz, C_4_-H_M_ pyrazoline), 5.59 (1H, dd, *J_MX_* = 11.88 Hz, *J_AX_* = 4.74 Hz, C_5_-H_X_ pyrazoline), 6.64 (1H, dd, *J*= 3.39 Hz, 1.77 Hz, aromatic proton), 6.89 (1H, d, *J* = 3.39 Hz, aromatic proton), 7.07 (2H, d, *J* = 8.88 Hz, aromatic protons), 7.14 (4H, s, aromatic protons), 7.65 (2H, d, *J* = 8.97 Hz, aromatic protons), 7.86 (1H, t, *J* = 1.59 Hz, 1.20 Hz, 0.39 Hz, aromatic proton). ^13^C-NMR (75 MHz, DMSO-*d*_6_) δ (ppm): 21.10 (CH_3_), 43.45 (CH_2_), 44.62 (CH_3_), 61.98 (CH), 112.76 (2CH, d, *J* = 18.75 Hz), 113.67 (CH), 126.06 (2CH), 129.02 (2CH), 129.55 (CH), 129.85 (C), 130.20 (2CH), 137.46 (C), 139.63 (C), 142.64 (C), 145.45 (CH), 147.35 (2C, d, *J* = 8.25 Hz). Anal. Calcd. for C_21_H_20_N_2_O_3_S: C, 66.29; H, 5.30; N, 7.36; Found: C, 66.26; H, 5.34; N, 7.39. MS (ESI) (*m/z*): [M]^+^ 380.90, [M + H]^+^ 382.00.

*1-(4-Sulfonamidophenyl)-3-(2-furanyl)-5-(4-methylphenyl)-2-pyrazoline* (**3i**). Yield: 88%. M.p.: 139–140 °C. IR ν_max_ (cm^−^^1^): 3369.64, 3257.77 (N-H stretching), 3120.82, (aromatic C-H stretching), 2920.23, 2852.72 (aliphatic C-H stretching), 1589.34, 1504.48 (C=N and C=C stretching), 1413.82, 1373.32, 1325.10, 1305.81, 1149.57, 1095.57, 1002.98 (C-N, C-O stretching and aromatic C-H in plane bending), 883.40, 866.04, 815.89, 738.74 (aromatic C-H out of plane bending and C-S stretching). ^1^H-NMR (300 MHz, DMSO-*d*_6_) δ (ppm): 2.24 (3H, s, CH_3_), 3.05 (1H, dd, *J_AM_* = 17.46 Hz, *J_AX_* = 4.92 Hz, C_4_-H_A_ pyrazoline), 3.89 (1H, dd, *J_MA_* = 17.49 Hz, *J_MX_* = 12.00 Hz, C_4_-H_M_ pyrazoline), 5.57 (1H, dd, *J_MX_* = 11.94 Hz, *J_AX_* = 4.83 Hz, C_5_-H_X_ pyrazoline), 6.63 (1H, dd, *J* = 3.45 Hz, 1.80 Hz, aromatic proton), 6.85 (1H, d, *J* = 3.41 Hz, aromatic proton), 6.99–7.03 (4H, m, aromatic and NH_2_ protons), 7.12–7.13 (4H, m, aromatic protons), 7.57 (2H, d, *J* = 8.97 Hz, aromatic protons), 7.84 (1H, dd, *J* = 1.71 Hz, 0.66 Hz). ^13^C-NMR (75 MHz, DMSO-*d*_6_) δ (ppm): 21.08 (CH_3_), 43.33 (CH_2_), 62.06 (CH), 112.48 (2CH, d, *J* = 12.00 Hz), 126.12 (2CH), 127.59 (2CH), 130.11 (2CH), 133.51 (C), 137.35 (CH), 138.73 (2CH), 141.85 (C), 145.24 (2C), 146.22 (C), 147.47 (C). Anal. Calcd. for C_20_H_19_N_3_O_3_S: C, 62.97; H, 5.02; N, 11.02; Found: C, 62.94; H, 5.03; N, 11.05. MS (ESI) (*m/z*): [M]^+^ 381.90, [M + H]^+^ 382.90.

*1-Phenyl-3-(2-thienyl)-5-(1,3-benzodioxol-5-yl)-2-pyrazoline* (**4a**) [44]. Yield: 83%. M.p.: 179–180 °C. IR ν_max_ (cm^−^^1^): 3105.39, 3070.68 (aromatic C-H stretching), 2916.37 (aliphatic C-H stretching), 1593.20, 1498.69, 1481.33, 1442.75 (C=N and C=C stretching), 1379.10, 1317.38, 1238.30, 1109.07, 1037.70 (C-N, C-O stretching and aromatic C-H in plane bending), 937.40, 823.60, 748.38, 719.45 (aromatic C-H out of plane bending and C-S stretching). ^1^H-NMR (300 MHz, DMSO-*d*_6_) δ (ppm): 3.10 (1H, dd, *J_AM_* = 17.37 Hz, *J_AX_* = 6.33 Hz, C_4_-H_A_ pyrazoline), 3.87 (1H, dd, *J_MA_* = 17.37 Hz, *J_MX_* = 12.09 Hz, C_4_-H_M_ pyrazoline), 5.40 (1H, dd, *J_MX_* = 12.00 Hz, *J_AX_* = 6.30 Hz, C_5_-H_X_ pyrazoline), 5.97 (2H, d, *J* = 0.57 Hz, O-CH_2_-O), 6.70–6.79 (3H, m, aromatic protons), 6.87 (1H, d, *J* = 8.13 Hz, aromatic proton), 6.95 (2H, d, *J* = 7.89 Hz, aromatic protons), 7.08–7.18 (3H, m, aromatic protons), 7.25 (1H, d, *J* = 3.51 Hz, aromatic proton), 7.59 (1H, d, *J* = 5.04 Hz, aromatic proton). ^13^C-NMR (75 MHz, DMSO-*d*_6_) δ (ppm): 44.18 (CH_2_), 63.51 (CH), 101.52 (CH_2_), 106.54 (CH), 109.07 (CH), 113.46 (2CH), 119.16 (CH), 119.57 (CH), 127.90 (2CH), 128.28 (CH), 129.35 (2CH), 136.15 (C), 136.59 (C), 144.21 (C), 144.50 (C), 146.99 (C), 148.19 (C). Anal. Calcd. for C_20_H_16_N_2_O_2_S: C, 68.95; H, 4.63; N, 8.04; Found: C, 68.93; H, 4.64; N, 8.05. MS (ESI) (*m/z*): [M + H]^−^ 346.90, [M]^+^ 347.90, [M + H]^+^ 348.90.

*1-(4-Cyanophenyl)-3-(2-thienyl)-5-(1,3-benzodioxol-5-yl)-2-pyrazoline* (**4b**). Yield: 93%. M.p.: 156–157 °C. IR ν_max_ (cm^−^^1^): 3105.39 (aromatic C-H stretching), 2916.37, 2848.86 (aliphatic C-H stretching), 2208.49 (C≡N stretching), 1600.92, 1510.26, 1481.33, 1442.75 (C=N and C=C stretching), 1396.46, 1325.10, 1242.16, 1174.65, 1095.57, 1035.77 (C-N, C-O stretching and aromatic C-H in plane bending), 935.48, 840.96, 821.68, 802.39, 727.16 (aromatic C-H out of plane bending and C-S stretching). ^1^H-NMR (300 MHz, DMSO-*d*_6_) δ (ppm): 3.20 (1H, dd, *J_AM_* = 17.64 Hz, *J_AX_* = 4.98 Hz, C_4_-H_A_ pyrazoline), 3.95 (1H, dd, *J_MA_* = 17.64 Hz, *J_MX_* = 11.94 Hz, C_4_-H_M_ pyrazoline), 5.57 (1H, dd, *J_MX_* = 11.82 Hz, *J_AX_* = 4.92 Hz, C_5_-H_X_ pyrazoline), 5.99 (2H, d, *J* = 1.71 Hz, O-CH_2_-O), 6.73 (1H, dd, *J* = 7.98 Hz, 1.71 Hz, aromatic proton), 6.77 (1H, d, *J* = 1.56 Hz, aromatic proton), 6.87 (1H, d, *J* = 7.95 Hz, aromatic proton), 7.01 (2H, d, *J* = 8.91 Hz, aromatic protons), 7.14 (1H, dd, *J* = 5.04 Hz, 3.66 Hz, aromatic proton), 7.36 (1H, dd *J* = 3.60 Hz, 1.05 Hz, aromatic proton), 7.57 (2H, d, *J* = 8.94 Hz, aromatic protons), 7.69 (1H, dd, *J* = 5.04 Hz, 1.02 Hz, aromatic proton). ^13^C-NMR (75 MHz, DMSO-*d*_6_) δ (ppm): 44.26 (CH_2_), 62.52 (CH), 99.45 (C), 101.65 (CH_2_), 106.41 (CH), 109.20 (CH), 113.21 (2CH), 119.42 (CH), 120.42 (C), 128.48 (CH), 129.18 (CH), 129.47 (CH), 133.80 (2CH), 135.24 (C), 135.46 (C), 146.75 (C), 147.24 (C), 147.51 (C), 148.33 (C). Anal. Calcd. for C_21_H_15_N_3_O_2_S: C, 67.54; H, 4.05; N, 11.25; Found: C, 67.56; H, 4.04; N, 11.24. MS (ESI) (*m/z*): [M]^+^ 373.90.

*1-(4-Fluorophenyl)-3-(2-thienyl)-5-(1,3-benzodioxol-5-yl)-2-pyrazoline* (**4c**). Yield: 76%. M.p.: 108–110 °C. IR ν_max_ (cm^−^^1^): 3086.11 (aromatic C-H stretching), 2970.38, 2916.37 (aliphatic C-H stretching), 1504.48, 1483.26, 1442.75 (C=N and C=C stretching), 1240.23, 1220.94, 1035.77 (C-N, C-O stretching and aromatic C-H in plane bending), 931.62, 802.39, 705.95 (aromatic C-H out of plane bending and C-S stretching). ^1^H-NMR (300 MHz, DMSO-*d*_6_) δ (ppm): 3.11 (1H, dd, *J_AM_* = 17.31 Hz, *J_AX_* = 6.81 Hz, C_4_-H_A_ pyrazoline), 3.87 (1H, dd, *J_MA_* = 17.31 Hz, *J_MX_* = 11.97 Hz, C_4_-H_M_ pyrazoline), 5.36 (1H, dd, *J_MX_* = 11.91 Hz, *J_AX_* = 6.75 Hz, C_5_-H_X_ pyrazoline), 5.98 (2H, d, *J* = 0.78 Hz, O-CH_2_-O), 6.77–6.81 (2H, m, aromatic protons), 6.87 (1H, d, *J* = 7.83 Hz, aromatic proton), 6.91–6.94 (2H, m, aromatic protons), 6.95–7.02 (2H, m, aromatic protons), 7.04–7.11 (1H, m, aromatic proton), 7.24–7.26 (1H, m, aromatic proton), 7.60 (1H, dd, *J* = 5.07 Hz, 1.05 Hz, aromatic proton). ^13^C-NMR (75 MHz, DMSO-*d*_6_) δ (ppm): 44.36 (CH_2_), 64.11 (CH), 101.55 (CH_2_), 106.62 (CH), 109.08 (CH), 114.65 and 114.75 (2CH), 115.75 and 116.05 (2CH), 119.70 (2CH), 127.98 and 128.28 (2CH), 136.04 and 136.31 (2C), 141.49 (C), 144.46 (C), 147.06 (C), 148.22 (C), 154.85 and 157.96 (C). Anal. Calcd. for C_20_H_15_FN_2_O_2_S: C, 65.56; H, 4.13; N, 7.65; Found: C, 65.54; H, 4.14; N, 7.66. MS (ESI) (*m/z*): [M + H]^−^ 364.90, [M]^+^ 365.90, [M + H]^+^ 366.90.

*1-(4-Chlorophenyl)-3-(2-thienyl)-5-(1,3-benzodioxol-5-yl)-2-pyrazoline* (**4d**). Yield: 92%. M.p.: 130–132 °C. IR ν_max_ (cm^−^^1^): 3072.60 (aromatic C-H stretching), 2916.37, 2848.86 (aliphatic C-H stretching), 1595.13, 1492.90, 1481.33, 1442.75 (C=N and C=C stretching), 1382.96, 1319.31, 1244.09, 1136.07, 1093.64, 1037.70 (C-N, C-O stretching and aromatic C-H in plane bending), 933.55, 813.96, 796.60, 725.23, 707.88 (aromatic C-H out of plane bending and C-S stretching). ^1^H-NMR (300 MHz, DMSO-*d*_6_) δ (ppm): 3.13 (1H, dd, *J_AM_* = 17.40 Hz, *J_AX_* = 6.09 Hz, C_4_-H_A_ pyrazoline), 3.89 (1H, dd, *J_MA_* = 17.43 Hz, *J_MX_* = 12.03 Hz, C_4_-H_M_ pyrazoline), 5.42 (1H, dd, *J_MX_* = 11.94 Hz, *J_AX_* = 6.03 Hz, C_5_-H_X_ pyrazoline), 5.98 (2H, d, *J* = 1.59 Hz, O-CH_2_-O), 6.74–6.78 (2H, m, aromatic protons), 6.87 (1H, d, *J* = 7.89 Hz, aromatic proton), 6.94 (2H, d, *J* = 9.03 Hz, aromatic protons), 7.11 (1H, dd, *J* = 5.07 Hz, 3.63 Hz, aromatic proton), 7.20 (2H, d, *J* = 9.00 Hz, aromatic protons), 7.27 (1H, dd, *J* = 3.57 Hz, 1.08 Hz, aromatic proton), 7.62 (1H, dd, *J* = 5.07 Hz, 1.05 Hz, aromatic proton). ^13^C-NMR (75 MHz, DMSO-*d*_6_) δ (ppm): 44.30 (CH_2_), 63.41 (CH), 101.57 (CH_2_), 106.52 (CH), 109.10 (CH), 114.85 (2CH), 116.52 (C), 119.58 (CH), 122.70 (CH), 128.27 (2CH, d, *J* = 6.75 Hz), 129.21 (2CH, d, *J* = 6.75 Hz), 135.85 (C), 136.06 (C), 143.26 (C), 145.04 (C), 147.09 (C), 148.24 (C). Anal. Calcd. for C_20_H_15_ClN_2_O_2_S: C, 62.74; H, 3.95; N, 7.32; Found: C, 62.76; H, 3.94; N, 7.31. MS (ESI) (*m/z*): [M + H]^−−^ 378.80, [M]^+^ 380.80, [M + H]^+^ 381.90, [M + H]^++^ 382.80, [M + H]^+++^ 383.90.

*1-(4-Bromophenyl)-3-(2-thienyl)-5-(1,3-benzodioxol-5-yl)-2-pyrazoline* (**4e**). Yield: 90%. M.p.: 136–138 °C. IR ν_max_ (cm^−^^1^): 3070.68 (aromatic C-H stretching), 2916.37 (aliphatic C-H stretching), 1589.34, 1481.33, 1442.75 (C=N and C=C stretching), 1382.96, 1319.31, 1244.09, 1128.36, 1091.71, 1035.77 (C-N, C-O stretching and aromatic C-H in plane bending), 935.48, 812.03, 707.88 (aromatic C-H out of plane bending and C-S stretching). ^1^H-NMR (300 MHz, DMSO-*d*_6_) δ (ppm): 3.13 (1H, dd, *J_AM_* = 17.40 Hz, *J_AX_* = 5.91 Hz, C_4_-H_A_ pyrazoline), 3.88 (1H, dd, *J_MA_* = 17.43 Hz, *J_MX_* = 12.09 Hz, C_4_-H_M_ pyrazoline), 5.42 (1H, dd, *J_MX_* = 11.88 Hz, *J_AX_* = 5.85 Hz, C_5_-H_X_ pyrazoline), 5.98 (2H, d, *J* = 1.56 Hz, O-CH_2_-O), 6.73–6.77 (2H, m, aromatic protons), 6.83–6.94 (3H, m, aromatic protons), 7.10 (1H, dd, *J* = 4.89 Hz, 3.75 Hz, aromatic proton), 7.27–7.33 (3H, m, aromatic protons), 7.62 (1H, d, *J* = 4.95 Hz, aromatic proton). ^13^C-NMR (75 MHz, DMSO-*d*_6_) δ (ppm): 44.29 (CH_2_), 63.29 (CH), 101.57 (CH_2_), 106.51 (CH), 109.11 (CH), 110.32 (CH), 115.34 (2CH), 119.56 (CH), 128.31 (2CH, d, *J* = 5.25 Hz), 132.00 (2CH), 135.82 (C), 136.01 (2C), 143.57 (C), 145.13 (C), 147.10 (C), 148.24 (C). Anal. Calcd. for C_20_H_15_BrN_2_O_2_S: C, 56.22; H, 3.54; N, 6.56; Found: C, 56.24; H, 3.53; N, 6.55. MS (ESI) (*m/z*): [M + H]^—^ 426.80, [M]^+^ 427.80.

*1-(4-Methylphenyl)-3-(2-thienyl)-5-(1,3-benzodioxol-5-yl)-2-pyrazoline* (**4f**). Yield: 47%. M.p.: 153–155 °C. IR ν_max_ (cm^−^^1^): 3070.68 (aromatic C-H stretching), 2916.37, 2848.86 (aliphatic C-H stretching), 1593.20, 1481.33, 1442.75 (C=N and C=C stretching), 1382.96, 1319.31, 1244.09, 1130.29, 1091.71, 1035.77 (C-N, C-O stretching and aromatic C-H in plane bending), 933.55, 812.03, 802.39, 723.31, 707.88 (aromatic C-H out of plane bending and C-S stretching). ^1^H-NMR (300 MHz, DMSO-*d*_6_) δ (ppm): 2.16 (3H, s, CH_3_), 3.07 (1H, dd, *J_AM_* = 17.28 Hz, *J_AX_* = 6.57 Hz, C_4_-H_A_ pyrazoline), 3.84 (1H, dd, *J_MA_* = 17.25 Hz, *J_MX_* = 12.03 Hz, C_4_-H_M_ pyrazoline), 5.35 (1H, dd, *J_MX_* = 11.97 Hz, *J_AX_* = 6.54 Hz, C_5_-H_X_ pyrazoline), 5.97 (2H, d, *J* = 1.26 Hz, O-CH_2_-O), 6.76 (2H, d, *J* = 6.84 Hz, aromatic protons), 6.86 (3H, d, *J* = 8.64 Hz, aromatic protons), 6.97 (2H, d, *J* = 8.43 Hz, aromatic protons), 7.09 (1H, dd, *J* = 5.04 Hz, 3.63 Hz, aromatic proton), 7.22 (1H, dd, *J* = 3.54 Hz, 0.99 Hz, aromatic proton), 7.57 (1H, dd, *J* = 5.04 Hz, 0.99 Hz, aromatic proton). ^13^C-NMR (75 MHz, DMSO-*d*_6_) δ (ppm): 20.54 and 20.57 (CH_3_), 44.14 (CH_2_), 63.80 (CH), 101.50 (CH_2_), 106.57 (CH), 109.02 (CH), 113.65 (2CH), 119.63 (CH), 127.61 (CH), 127.88 (CH), 128.24 (CH), 129.78 (2CH), 136.29 (C), 136.63 (2C), 142.43 (C), 143.67 (C), 146.95 (C), 148.14 (C). Anal. Calcd. for C_21_H_18_N_2_O_2_S: C, 69.59; H, 5.01; N, 7.73; Found: C, 69.61; H, 5.00; N, 7.72. MS (ESI) (*m/z*): [M + H]^−−^ 360.90, [M]^+^ 361.90, [M + H]^+^ 362.90.

*1-(4-Methoxyphenyl)-3-(2-thienyl)-5-(1,3-benzodioxol-5-yl)-2-pyrazoline* (**4g**). Yield: 39%. M.p.: 140–142 °C. IR ν_max_ (cm^−^^1^): 3107.32 (aromatic C-H stretching), 2916.37, 2848.86 (aliphatic C-H stretching), 1587.42, 1504.48, 1487.12, 1444.68 (C=N and C=C stretching), 1375.25, 1230.58, 1180.44, 1116.78, 1083.99, 1033.85 (C-N, C-O stretching and aromatic C-H in plane bending), 935.48, 829.39, 804.32, 707.88 (aromatic C-H out of plane bending and C-S stretching). ^1^H-NMR (300 MHz, DMSO-*d*_6_) δ (ppm): 3.07 (1H, dd, *J_AM_* = 17.16 Hz, *J_AX_* = 7.41 Hz, C_4_-H_A_ pyrazoline), 3.65 (3H, s, OCH_3_), 3.83 (1H, dd, *J_MA_* = 17.19 Hz, *J_MX_* = 11.94 Hz, C_4_-H_M_ pyrazoline), 5.29 (1H, dd, *J_MX_* = 11.85 Hz, *J_AX_* = 7.38 Hz, C_5_-H_X_ pyrazoline), 5.98 (2H, d, *J* = 0.81 Hz, O-CH_2_-O), 6.76–6.81 (4H, m, aromatic protons), 6.85–6.91 (3H, m, aromatic protons), 7.09 (1H, dd, *J* = 5.04 Hz, 3.60 Hz, aromatic proton), 7.21 (1H, dd, *J* = 3.54 Hz, 1.02 Hz, aromatic proton), 7.56 (1H, dd, *J* = 5.04 Hz, 1.02 Hz, aromatic proton). ^13^C-NMR (75 MHz, DMSO-*d*_6_) δ (ppm): 44.24 (CH_2_), 55.64 and 55.67 (CH_3_), 64.66 (CH), 101.50 (CH_2_), 106.71 (CH), 109.01 (CH), 114.83 (2CH), 115.05 (2CH), 119.82 (CH), 127.46 (2CH, d, *J* = 6.00 Hz), 128.22 (CH), 136.37 (C), 136.63 (C), 139.11 (C), 143.49 (C), 146.97 (C), 148.14 (C), 153.25 (C). Anal. Calcd. for C_21_H_18_N_2_O_3_S: C, 66.65; H, 4.79; N, 7.40; Found: C, 66.67; H, 4.78; N, 7.39. MS (ESI) (*m/z*): [M]^+^ 377.90, [M + H]^+^ 378.90.

*1-(4-Methylsulfonylphenyl)-3-(2-thienyl)-5-(1,3-benzodioxol-5-yl)-2-pyrazoline* (**4h**). Yield: 63%. M.p.: 172–173 °C. IR ν_max_ (cm^−^^1^): 3097.68 (aromatic C-H stretching), 2985.81, 2900.84 (aliphatic C-H stretching), 1589.34, 1502.55, 1483.26, 1442.75 (C=N and C=C stretching), 1392.61, 1381.03, 1296.16, 1247.94, 1078.21, 1051.20 (C-N, C-O stretching and aromatic C-H in plane bending), 821.68, 769.60, 705.95 (aromatic C-H out of plane bending and C-S stretching). ^1^H-NMR (300 MHz, DMSO-*d*_6_) δ (ppm): 3.07 (3H, s, SO_2_CH_3_), 3.20 (1H, dd, *J_AM_* = 17.64 Hz, *J_AX_* = 5.07 Hz, C_4_-H_A_ pyrazoline), 3.96 (1H, dd, *J_MA_* = 17.61 Hz, *J_MX_* = 11.94 Hz, C_4_-H_M_ pyrazoline), 5.57 (1H, dd, *J_MX_* = 11.85 Hz, *J_AX_* = 4.98 Hz, C_5_-H_X_ pyrazoline), 5.98 (2H, d, *J* = 1.62 Hz, O-CH_2_-O), 6.75 (1H, dd, *J* = 7.95 Hz, 1.74 Hz, aromatic proton), 6.79 (1H, d, *J* = 1.56 Hz, aromatic proton), 6.88 (1H, d, *J* = 7.92 Hz, aromatic proton), 7.07 (2H, d, *J* = 8.94 Hz, aromatic protons), 7.13 (1H, dd, *J* = 5.07 Hz, 3.66 Hz, aromatic proton), 7.36 (1H, dd, *J* = 3.57 Hz, 1.05 Hz, aromatic proton), 7.65–7.69 (3H, m, aromatic protons). ^13^C-NMR (75 MHz, DMSO-*d*_6_) δ (ppm): 44.29 (CH_2_), 44.63 (CH_3_), 62.61 (CH), 101.64 (CH_2_), 106.43 (CH), 109.21 (CH), 112.61 (2CH), 119.45 (CH), 128.47 (CH), 129.05 (2CH), 129.38 (2CH), 129.61 (C), 135.30 (C), 135.56 (2C), 147.27 (2C), 148.32 (C). Anal. Calcd. for C_21_H_18_N_2_O_4_S_2_: C, 59.14; H, 4.25; N, 6.57; Found: C, 59.16; H, 4.24; N, 6.56. MS (ESI) (*m/z*): [M]^+^ 426.80, [M + H]^+^ 427.80.

*1-(4-Sulfonamidophenyl)-3-(2-thienyl)-5-(1,3-benzodioxol-5-yl)-2-pyrazoline* (**4i**) [45]. Yield: 83%. M.p.: 129–130 °C. IR ν_max_ (cm^−^^1^): 3321.42, 3250.05 (N-H stretching), 3103.46, 3074.53 (aromatic C-H stretching), 2916.37, 2848.86 (aliphatic C-H stretching), 1591.27, 1502.55, 1485.19, 1442.75 (C=N and C=C stretching), 1394.53, 1323.17, 1307.74, 1238.30, 1151.10, 1095.57, 1035.77, 1001.06 (C-N, C-O stretching and aromatic C-H in plane bending), 933.55, 904.61, 860.25, 812.03, 713.66 (aromatic C-H out of plane bending and C-S stretching). ^1^H-NMR (300 MHz, DMSO-*d*_6_) δ (ppm): 3.18 (1H, dd, *J_AM_* = 17.52 Hz, *J_AX_*= 5.10 Hz, C_4_-H_A_ pyrazoline), 3.93 (1H, dd, *J_MA_* = 17.52 Hz, *J_MX_* = 11.94 Hz, C_4_-H_M_ pyrazoline), 5.56 (1H, dd, *J_MX_* = 11.85 Hz, *J_AX_* = 5.01 Hz, C_5_-H_X_ pyrazoline), 5.98 (2H, d, *J* = 2.04 Hz, O-CH_2_-O), 6.72–6.77 (2H, m, aromatic protons), 6.87 (1H, d, *J* = 7.86 Hz, aromatic proton), 7.01–7.04 (4H, m, aromatic and NH_2_ protons), 7.12 (1H, dd, *J* = 5.04 Hz, 3.63 Hz, aromatic proton), 7.33 (1H, dd, *J* = 3.57 Hz, 1.05 Hz, aromatic proton), 7.60 (2H, d, *J* = 8.94 Hz, aromatic protons), 7.66 (1H, dd, *J* = 5.04 Hz, 1.02 Hz, aromatic proton). ^13^C-NMR (75 MHz, DMSO-*d*_6_) δ (ppm): 44.83 (CH_2_), 64.86 (CH), 101.66 (CH_2_), 106.85 (CH), 109.87 (CH), 115.65 (2CH), 119.87 (CH), 127.47 (CH), 127.96 (CH), 128.05 (CH), 131.88 (2CH), 135.77 (C), 136.45 (C), 146.03 (C), 147.49 (C), 148.90 (C), 159.36 (2C). Anal. Calcd. for C_20_H_17_N_3_O_4_S_2_: C, 56.19; H, 4.01; N, 9.83; Found: C, 56.24; H, 4.08; N, 9.80. MS (ESI) (*m/z*): [M]^+^ 427.80, [M + H]^+^ 428.80.

### 3.2. Pharmacology

#### 3.2.1. Cell Culture

PC-12 Adh cells were cultured in 10% horse serum, 5% fetal bovine serum, and 1% penicillin-streptomycin containing DMEM growth medium, at 37 °C in a humidified incubator with 5% CO_2_. The proliferating cells were passaged 1:2 to new 25 and/or 75 cm^2^ flasks and cell stocks were prepared in order to use in future experiments. In order to induce PC-12 Adh cell differentiation into neuronal phenotype, growth medium was changed with 1% fetal bovine serum, 1% penicillin-streptomycin and 100 nM NGF containing DMEM differentiation medium.

Differentiated PC-12 Adh cells were stained with Trypan blue solution and counted by a cell counter device (Cedex XS, Innovatis, Malvern, PA, USA) in order to determine the appropriate cell numbers before the experiments.

#### 3.2.2. Determination of Non-Cytotoxic Concentrations

In order to obtain non-cytotoxic concentrations of the compounds, the viability of neuronal cells was measured by using 2-(4-iodophenyl)-3-(4-nitrophenyl)-5-(2,4-disulfophenyl)-2*H*-tetrazolium sodium salt (WST-1) assay (Roche, Mannheim, Germany). The test is based on the cleavage of the tetrazolium salt WST-1 in formazan by mitochondrial dehydrogenases in viable cells. The formazan dye was quantified in a scanning multiwell spectrophotometer by measuring the absorbance of the dye at 420 nm. The differentiated PC-12 Adh cells were scratched and plated onto 96-well culture plates at 5 × 10^3^ density per well. After 24 h, the cells were treated with 400, 200, 100, 50, and 25 µg/mL concentrations of compounds and 400, 200, 100, 50 and 25 µM 6-OHDA for 24 h. After the incubation period, the cell proliferation reagent WST-1 (10 μL per well) was added to the wells; and absorbances were measured after 3 h using a Cytation 3 cell imaging multi-mode reader at 420 nm (BioTek, Winooski, VT, USA). The measured absorbances directly correlated to the number of viable cells. The cell viability rates were expressed as a percentage of the controls, and IC_50_ values of the compounds and 6-OHDA were calculated according to the control group [46].

#### 3.2.3. Determination of Neuroprotective Activity against 6-OHDA-Induced Neurodegeneration

In order to determine the in vitro neuroprotective potentials of the compounds, 6-OHDA-induced neurotoxicity model of PD was conducted [47,48]. The differentiated PC-12 Adh cells were scratched and plated onto 96-well culture plates at 5 × 10^3^ density per well. After 24 h, the cells were treated with 100 µg/mL concentration (only 10 µg/mL and 100 µg/mL were tested for compound **1**, which was found to be highly cytotoxic) of the compounds for 6 h. 100 µg/mL concentration of the compounds was determined as the non-cytotoxic concentration, and the IC_50_ value of the 6-OHDA (150 µM) was used as a positive control according to the WST-1 cell viability assay results. After a 6-h incubation period with the compounds, the medium was removed, and the cells were treated with 150 µM 6-OHDA in order to induce neurodegeneration via oxidative stress for 24 h. The control cells were cultured in differentiation medium containing 0.1% DMSO, and the cells cultured in 150 µM 6-OHDA were used as a positive control. After 24 h, the neuroprotective effects of the compounds were determined by WST-1 cell viability assay as explained above. The graphics were drawn according to the cell viability, which was expressed as percentage of the surviving control cells in the study.

#### 3.2.4. Statistical Analysis

The graphics were drawn with Graphpad Prism 6.0 software and statistically analyzed using one-way ANOVA and Tukey’s post hoc test. The results are expressed as mean ± standard deviation and the means of three independent experiments (*n* = 8), n.s; *p* > 0.05, * *p* < 0.05, ** *p* < 0.01, *** *p* < 0.001, and **** *p* < 0.0001 were considered significant compared with the control group and the 6-OHDA-positive control group.

### 3.3. Prediction of Pharmacokinetic Parameters

ADME properties of compounds **1**, **2**, **3a**–**i**, and **4a**–**i** were in silico predicted using QikProp program (Schrödinger Release 2016-2: QikProp, Schrödinger, LLC, New York, NY, USA, 2016). This program computes physically significant descriptors and pharmaceutically relevant properties, such as QPlogBB, CNS activity, SASA (in square angstroms using a probe with a 1.4 Å Radius) and a percentage of human oral absorption. The acceptability of compounds **1**, **2**, **3a**–**i** and **4a**–**i**, based on the Lipinski’s rule of five [39] and Jorgensen’s rule of three [40], was also determined.

## 4. Conclusions

In the recent work, 2-pyrazoline based compounds **3a**–**i** and **4a**–**i** were synthesized based on the reaction of chalcones (**1**, **2**) and phenylhydrazine hydrochloride derivatives. All these compounds were evaluated for their neuroprotective effects against toxicity induced by 6-OHDA in rat pheochromocytoma (PC-12) cells. According to in vitro studies, 4-methylsulfonylphenyl containing compounds **3h** and **4h** induced cell viability percentage notably when compared with the 6-OHDA-positive control group. In silico ADME prediction also pointed out that all compounds were within the acceptable range for some pharmacokinetic parameters especially important for CNS activity. Consequently, compounds **3h** and **4h** stand out as potential orally bioavailable CNS acting drug candidates for further neuroprotective studies associated with PD.

## Figures and Tables

**Table 1:** IC_50_ values of compounds **1**, **2**, **3a**–**i** and **4a**–**i** and 6-OHDA according to the cell viability assay.

Compound	IC_50_ (µg/mL)	Compound	IC_50_ (µg/mL)
**1**	12	**2**	345
**3a**	>400	**4a**	>400
**3b**	204	**4b**	>400
**3c**	353	**4c**	>400
**3d**	>400	**4d**	>400
**3e**	398	**4e**	>400
**3f**	>400	**4f**	239
**3g**	>400	**4g**	252
**3h**	>400	**4h**	>400
**3i**	205	**4i**	>400
6-OHDA	150 µM	6-OHDA	150 µM

**Table 2:** Cell viability values of 6-OHDA and compounds **1**, **2**, **3a**–**i**, **4a**–**i** according to the cell viability assay results. The results of the cell viability were presented as a % of the control (the O.D. value).

Compound	Cell Viability %	Compound	Cell Viability %
Control	100	Control	100
6-OHDA	57	6-OHDA	58
**1** (100 µg/mL)	3	**2**	66
**1** (10 µg/mL)	60	**4a**	41
**3a**	64	**4b**	46
**3b**	71	**4c**	24
**3c**	65	**4d**	44
**3d**	63	**4e**	59
**3e**	70	**4f**	38
**3f**	69	**4g**	31
**3g**	71	**4h**	77
**3h**	80	**4i**	13
**3i**	70		

**Table 3:** Predicted ADME properties of compounds **1**, **2**, **3a**–**i**, and **4a**–**i**.

Compound	QplogBB * (−3 to 1.2)	CNS * (−2 to 2)	SASA * (300.0 to 1000.0)	Human Oral Absorption% * (>80% Is High, <25% Is poor)	Rule of Five **	Rule of Three ***
**1**	−0.12	0	482	100	0	0
**2**	0.02	1	467	100	0	0
**3a**	0.49	2	594	100	1	1
**3b**	−0.31	0	632	100	0	1
**3c**	0.60	2	603	100	1	1
**3d**	0.66	2	618	100	1	1
**3e**	0.67	2	623	100	1	1
**3f**	0.48	2	626	100	1	1
**3g**	0.42	1	631	100	1	1
**3h**	−0.40	0	680	100	0	1
**3i**	−1.08	−2	666	93	0	1
**4a**	0.59	2	582	100	1	1
**4b**	−0.16	0	620	100	0	1
**4c**	0.71	2	591	100	1	1
**4d**	0.77	2	606	100	1	1
**4e**	0.78	2	611	100	1	1
**4f**	0.59	2	614	100	1	1
**4g**	0.53	2	619	100	1	1
**4h**	−0.26	0	669	100	0	0
**4i**	−0.91	−1	654	92	0	0

* QPlogBB: brain/blood partition coefficient, CNS: predicted central nervous system activity, SASA: total solvent accessible surface area, in square angstroms using a probe with a 1.4 Å Radius, and Percent Human Oral Absorption: human oral absorption on a 0–100% scale. ** Rule of Five: Number of violations of Lipinski’s rule of five. The rules are as follows: mol_MW (molecular weight of the molecule) < 500, QPlogPo/w (predicted octanol/water partition coefficient) < 5, donorHB (hydrogen-bond donor atoms) ≤ 5, and accptHB (hydrogen-bond acceptor atoms) ≤ 10. Compounds that provide these rules are considered as drug-like molecules. *** Rule of Three: Number of violations of Jorgensen’s rule of three. The three rules are as follows: QPlogS (predicted aqueous solubility) > −5.7, QPPCaco (predicted apparent Caco-2 cell permeability in nm/s) > 22 nm/s, Primary Metabolites < 7. Compounds with fewer (and preferably no) violations of these rules are more likely to be orally available (Schrödinger Release 2016-2: QikProp, Schrödinger, LLC, New York, NY, 2016).
